# Physically-Induced Cytoskeleton Remodeling of Cells in Three-Dimensional Culture

**DOI:** 10.1371/journal.pone.0045512

**Published:** 2012-12-27

**Authors:** Sheng-Lin Lee, Ali Nekouzadeh, Boyd Butler, Kenneth M. Pryse, William B. McConnaughey, Adam C. Nathan, Wesley R. Legant, Pascal M. Schaefer, Robert B. Pless, Elliot L. Elson, Guy M. Genin

**Affiliations:** 1 Department of Mechanical Engineering & Materials Science, Washington University, St. Louis, Missouri, United States of America; 2 Department of Biomedical Engineering, Washington University, St. Louis, Missouri, United States of America; 3 Department of Biological Sciences Texas Tech University, Lubbock, Texas, United States of America; 4 Department of Biochemistry & Molecular Biophysics, Washington University School of Medicine, St. Louis, Missouri, United States of America; 5 Department of Biology, Washington University, St. Louis, Missouri, United States of America; 6 Department of Computer Science and Engineering, Washington University, St. Louis, Missouri, United States of America; University of Rochester, United States of America

## Abstract

Characterizing how cells in three-dimensional (3D) environments or natural tissues respond to biophysical stimuli is a longstanding challenge in biology and tissue engineering. We demonstrate a strategy to monitor morphological and mechanical responses of contractile fibroblasts in a 3D environment. Cells responded to stretch through specific, cell-wide mechanisms involving staged retraction and reinforcement. Retraction responses occurred for all orientations of stress fibers and cellular protrusions relative to the stretch direction, while reinforcement responses, including extension of cellular processes and stress fiber formation, occurred predominantly in the stretch direction. A previously unreported role of F-actin clumps was observed, with clumps possibly acting as F-actin reservoirs for retraction and reinforcement responses during stretch. Responses were consistent with a model of cellular sensitivity to local physical cues. These findings suggest mechanisms for global actin cytoskeleton remodeling in non-muscle cells and provide insight into cellular responses important in pathologies such as fibrosis and hypertension.

## Introduction

How cells respond to alterations in mechanical forces is critical in homeostasis, in tissue development [1]–[3] and in pathologies such as cardiomyopathy [4] and asthma [5]–[13]. These responses are particularly important in cells that serve a mechanical function, such as myofiboblsts, a unique group of smooth muscle-like fibroblasts that play an important role in oncogenesis, inflammation, wound contraction and fibrosis. Fibroblasts transition into the myofibroblastic phenotype in response to environmental factors including mechanical stress, transforming growth factor β (TGF-β), and the ED-A splice variant of fibronectin [14], [15].

Studies of living cells cultured on two-dimensional (2D) substrata have revealed a range of mechanical and structural responses of cells to mechanical perturbation. Mechancially-induced cellular responses include cytoskeletal fluidization in response to a single stretch followed by a release [16], [17], and cytoskeletal reinforcement in response to a single sustained stretch [17] or localized stressing [16]. These cytoskeletal changes and associated reorientation of cellular tractions occur over timescales much shorter than those associated with reorientation of a cell body on a 2D substratum [18].

**Figure 1:**
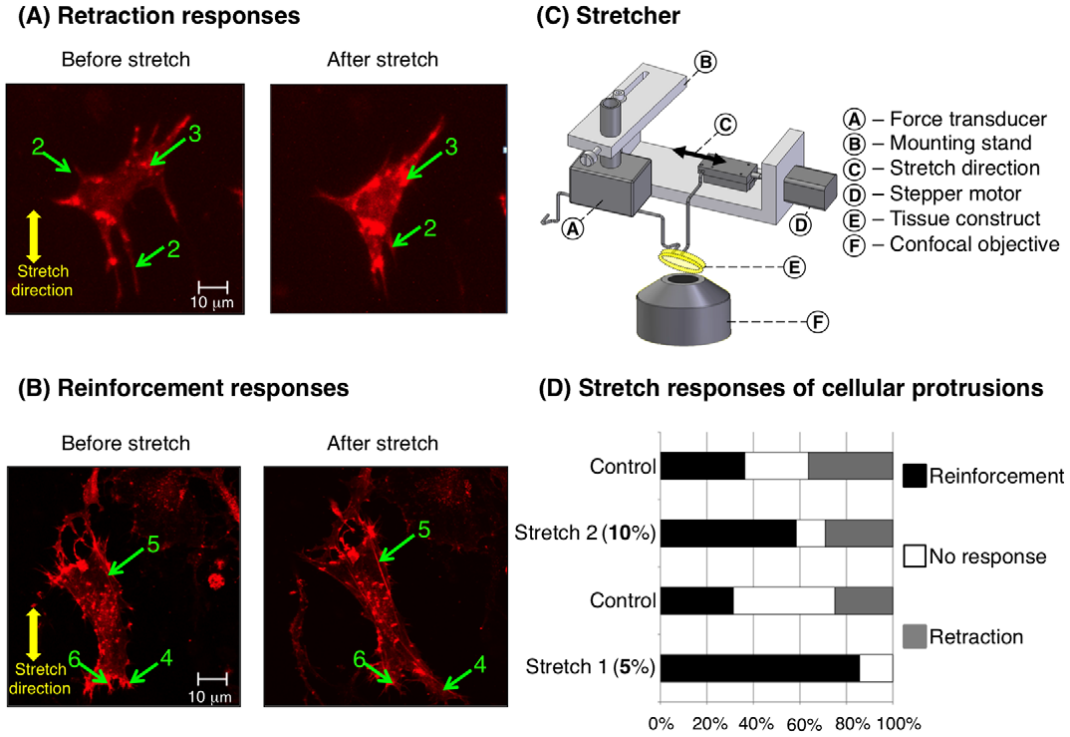
Responses of contractile fibroblasts to mechanical stretch. A fraction of contractile fibroblasts in a 3D tissue construct were transfected with mCherry-LifeAct (red) prior to being mixed with collagen and incubated for three days. Cells are shown after preconditioning (A and B, left), and then in a stretched state (A and B, right), 30 minutes after rapid application of a sustained 5% or 10% stretch using the device shown in (C) (see also movie S3 and S4). Cells exhibited two classes of responses to mechanical stimuli. Retraction responses (A) involved retraction of filopodium-like cellular protrusions (labels 2), and formation and growth of F-actin reservoirs (label 3), often near the site of retraction of cellular protrusions or stress fiber depolymerization. Reinforcement responses involved polymerization and thickening of actin stress fibers (label 5), extension of cellular protrusions (label 4), and decreases in the sizes and numbers of F-actin reservoirs (label 6), often near the site of the extension of cellular protrusions or growth of stress fibers (D). Stretch increased the likelihood that cellular protrusions would extend, and decreased the likelihood that they would retract; however, only the former was significant statistically.

A central question is whether these observations of cells cultured on 2D substrata are representative of behaviors that these same cells would exhibit in a natural tissue environment *in vivo*. We have addressed this by developing 3D engineered tissue constructs (ETCs) as model systems in which to probe cellular biophysical properties. In earlier results, cytoskeletal fluidization (deploymerization) and reinforcement (polymerization) following rapidly-applied, sustained stretch were observed in cells within 3D ETCs [19]. However, the time courses and mechanisms by which these responses occur have not been established previously for living cells in 3D culture. To elucidate these, we developed a system to stretch and then hold ETCs over the objective of a confocal fluorescence microscope (Figure 1C). Using this system, we observed and quantified cytoskeletal dynamics in a fraction of cells within ETCs that were transfected with m-cherry LifeAct, a fluorescent F-actin reporter.

Results showed that myofibroblasts can respond to stretch through specific, cell-wide mechanisms involving staged retraction and reinforcement. Retraction responses occurred for all orientations of stress fibers and cellular protrusions relative to the stretch direction, while reinforcement responses, including extension of cellular processes and stress fiber formation, occurred predominantly in the stretch direction. A previously unreported role of F-actin clumps was observed, with these clumps acting as F-actin reservoirs for retraction and reinforcement responses during stretch. In the following, we present these data and propose mechanical mechanisms underlying these responses through a simplified mathematical model of cellular sensitivity to local physical cues.

## Methods

### Preparation of 3D engineered tissue constructs

Chicken embryo fibroblast (CEF)-populated ETCs were synthesized to provide a realistic 3D collagen-based environment in which to probe effects of mechanical stimulus on cytoskeletal dynamics. CEFs isolated from 10-day chicken embryos (fertile white leghorn chicken eggs, Sunrise Farms, Catskill, NY) were maintained in Dulbecco's modified Eagle's medium (DMEM) with 10% fetal calf serum (FCS), penicillin and streptomycin at 50 units/ml and 50 μg/ml respectively, and without phenol red, a pH indicator. This solution was combined with home-made monomeric type I rat tail collagen dissolved in 0.02 M acetic acid, neutralized if necessary at 4°C with 0.1 M NaOH. Concentrated DMEM was also added to yield a final collagen content of 1 mg/ml. For some experiments fluorescent carboxylate microspheres (#17156, Polysciences Inc., Warrington, PA), 5.7 μm diameter, were added to the cell/collagen suspension at a final concentration of 3.55×10^3^ particles/ml. In all experiments, 5–10% of cells were transfected with the F-actin reporter m-cherry LifeAct. The LifeAct peptide associates with actin filaments without affecting cellular dynamics [20]. Neutralized solutions were composed of collagen (1.0 mg/ml) with a cell density of 10^6^ cells/ml. The solution was poured into annular molds (7.92 mm inner diameter, 19.0 mm outer diameter) [21]. After three days of culture, over which the cells actively remodeled and compressed the collagen solution, ring-shaped ETCs were removed from the inner mandrels of the molds and mounted on the custom designed stretcher for testing (Figure 1). To ensure that the transfection was not toxic to cells, comparisons were made between populations of transfected and untransfected cultures over periods of 5 days; no significant trends in proliferation or viability were observed relative to controls.

### Cell stretching system

To simultaneously measure ETC force responses and monitor cellular responses inside uniaxially stretched ETCs, a custom-made tissue stretcher was designed to work over a confocal microscope (Figure 1C). The stretcher is described in detail elsewhere [22]. Briefly, the stretcher consisted of three major subassemblies: an isometric force transducer, a mounting stand, and a linear actuating stepper motor. The isometric force transducer (model 724490, Harvard Apparatus, Holliston, MA) connected to the one end of the tissue through a titanium mounting rod, and the linear actuating stepper motor to the other through a second titanium mounting rod. The linear actuating stepper motor has a resolution of 1.5 μm per step. To make the device capable of moving with a tolerance of less than 1.0 μm, an eighth-stepping driver (custom developed by Gavin Perry, Washington University Electronics Shop) was used. We used a computer with custom-made software in the Experix environment (http://sourceforge.net/projects/experix/) operating on a Linux-based system to send signals through the digital I/O channels of a PCMCIA card (model DAS26/16-AO, Measurement Computing, Norton, MA) to the stepper motor via the eighth-stepping driver. The software also acquired force data from the force transducer through the same card. Scripts were written for two stretching protocols: a cyclic stretch, used for preconditioning, and a rapid step stretch followed by an isometric hold, in which tissue constructs were lengthened at a strain rate of approximately 60%/s then held at that length while the force was monitored for 30 minutes.

### Experimental protocols

The mechanical and morphological responses of cells to stretch were measured. Following mounting of ring-shaped ETCs onto the stretcher, the ETC and stretcher were placed over the objective of a Ziess confocal laser scanning microscope (Axiovert 200 M with LSM 510 ConfoCor 2, Carl Zeiss Inc., Oberkochen, Germany) with fluorescence excited by a helium-neon laser. The confocal fluorescence microscope was focused to locate stained cells within the ETC to monitor dynamic activity before and following stretching of the ETC. The stretching protocol was a series of 10% preconditioning stretches applied at 20%/s, followed by 30 minute relaxation period, then a 10% stretch at 60%/s and an isometric hold for thirty minutes while force and imaging data were acquired. The first time point imaged in each case was approximately two minutes after stretch, allowing sufficient time for stretch-induced cytoskeletal depolymerization to occur. This 10% stretch was followed by a 30% stretch and an isometric hold for an additional 30 minute interval of force and imaging data acquisition. ETCs were kept at 37°C in on a heated micro-incubator stage.

When mounted over the loading arms, the ring-shaped ETCs adopted a rubber band-like shape. Tracking of fluorescent beads in the vicinity of the cells within the long planar faces of the rubber band-shaped ETCs revealed that the 10% and 30% ETC stretches resulted in local stretches of approximately 5% and 10%, respectively (supporting material, Figure S1). This was expected due to the anisotropy of the ETCs, which remodeled such that collagen fibrils and some cells preferentially aligned circumferentially. The result was that the ETCs are expected to be far more compliant radially than circumferentially, and over half of the displacement of the loading bars was absorbed by local, radial, compressive deformation at the points of application of force.

To better understand the dynamics of fibroblasts in response to external mechanical stimuli, we studied cellular responses in some ETCs treated with 10 µM Y-27632. Y-27632 has been widely used as a Rho-associated kinase (ROCK) inhibitor to identify and evaluate the involvement and roles of ROCK kinases in many biological phenomena, including wound healing, cardiomyocyte hypertrophy, and bronchial smooth muscle contraction [23]. To study effects of Ca^2+^, some specimens were treated with thapsigargin to release internal stores of Ca^2+^ [24], and then with BAPTA AM to chelate the internal Ca^2+^ and EGTA to chelate Ca^2+^ in the external medium.

### Microscopy and image processing

Multidimensional images were acquired using a long working distance 20X objective. All images were acquired with 10-bit resolution. The imaging protocol involved tracking the reconfiguration of cells with a stack of confocal microscopy images taken through the cell thickness every 5 minutes. The first time point imaged in each case was approximately two minutes after stretch, allowing sufficient time for stretch-induced cytoskeletal depolymerization to occur.

Images were processed using the FABLE algorithm to estimate the total contour length of stress fibers that appeared in each collapsed confocal z-stack of images [25]. The FABLE algorithm involves tracking changes over time in filtered 2D power spectral density of images. The filter was chosen to highlight features of the approximate thickness range of stress fibers. The normalized power in the band-pass filtered power spectral density scales with the total length of image features with the thickness range of stress fibers, and is termed “fibrosity.” Images were adjusted for net fluorescence prior to application of the FABLE algorithm to compensate for changes to total luminosity associated with stretch-induced motion of cells in the *z*-direction.

### Mathematical model

We developed and studied a mechanical model of a simplified cell to assess the hypothesis that responses to passive, local, physical cues could combine to produce the global cellular remodeling sequences observed in our experiments. The model is described in detail in Methods S1. The model was meant to be illustrative, and the illustration was limited to stress fibers. The extension of the model to filopodium-like cellular processes is straightforward. All parameter values were estimated from the literature, and no attempt was made to optimize parameters of the model to fit our specific experimental observations.

Cells were idealized as populations of stress fibers, adhered to and deforming in registry with an ECM. Cells and stress fibers exist in a state of tension, and each stress fiber was assigned a level of prestretch from within an allowable range [26], [27] that represented the degree to which each stress fiber was distended from its stress free configuration. The inputs to the model were a cell shape, a population of stress fibers with prescribed orientations and random levels of pre-stretch, and a local strain field resulting from mechanical stretch of an ETC. Outputs of the model were predicted behavior of stress fibers over time (polymerization and depolymerization) and an estimate of the fibrosity measure that would be observed experimentally. A flowchart of the model, specialized to the case of stress fibers, is shown in Figure S2 (supporting material).

Two basic principles governed stress fiber dynamics in the model. First was the widely-reported observation that stress fibers depolymerize with too great or with insufficient stretch (e.g. [26]–[37]). Second was a hypothesis that peak stress within an intermediate range at an adhesion site drives stress fiber polymerization towards a maximum allowable density over a specific time scale [38]. An underlying principle was that F-actin reservoirs provide a store of F-actin or possibly stress fiber fragments that could be recruited rapidly to form new stress fibers or cellular protrusions.

**Figure 2:**
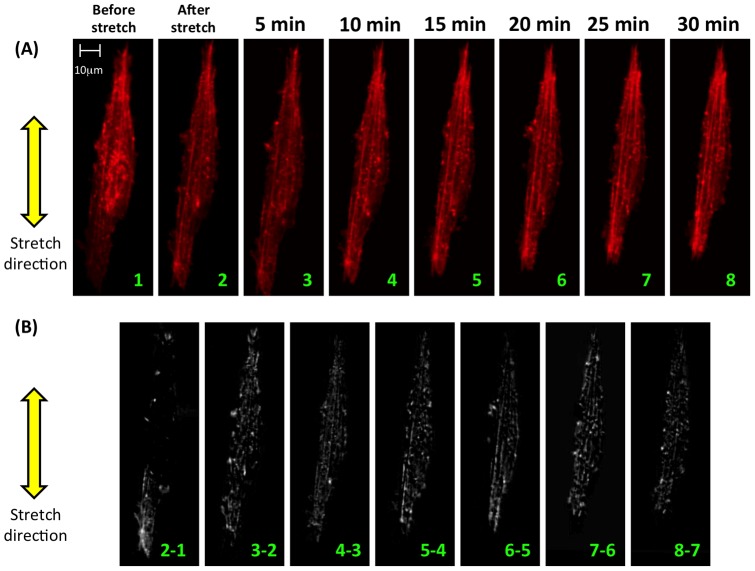
Time series (frame rate: one confocal image stack every five minutes) showing partial fluidization followed by reinforcement and formation of actin stress fibers in response to mechanical stretch of 5%. (A) Following stretch, the actin cytoskeleton fluidized partially, but stress fibers reappeared or were reinforced over the 30 minutes of monitoring. The cell pictured contracted as reinforcement occurred, shortening in length along the stretch direction. (B) Formation and reinforcement of stress fibers is evident from subtraction images involving differences in image intensities of subsequent images (e.g. “2–1” represents the subtraction of image 1 from image 2). Images were aligned prior to subtraction using standard techniques in Matlab. Note that subtraction images contain effects of both cell shortening and stress fiber formation.

We combined these basic principles into the first order mathematical model then studied the responses of idealized cells. Two limiting cases of pre-stretch cell morphology were explored, with similar results. The first limiting case represented the extreme of spindle-shaped cells such as that of Figure 2, with a clearly defined natural axis; the second represented cells such as that in Figure 1A, with no natural axis. Further details are available in Methods S1.

## Results

Prior to mechanical stretch most cells displayed diffuse cytoskeletons composed of randomly arranged actin filaments and aggregations of actin “reservoirs” (e.g. Figure 1A, label 3), and a few cells contained visible stress fibers (e.g. Figure 2 and Movie S1). Actin reservoirs in both transfected and non-transfected cells were shown by rhodamine phalloidin staining to contain F-actin in ETCs (Figure S3, supporting material). Immunostaining indicated that *α*-actinin was also present in analogous clumps (Figure S3, supporting material).

Cells in ETCs exhibited fliopodium-like cellular protrusions (e.g. Figure 1A, labels 2) into the extracellular matrix (ECM) that extended and retracted continuously (movie S2). In response to rapid stretch, the force necessary to maintain the ETCs isometrically increased from the baseline pre-stretch value (Figure S4, supporting material). The isometric force then decreased to slightly above the baseline pre-stretch value over approximately 30 minutes as cells and ECM relaxed viscoelastically and as cells remodeled actively.

### Cells in ETCs adapt actively to stretch

Cells exhibited staged responses to mechanical stretch (Figure 1, time courses in movie S3 and S4). Retraction responses involved depolymerization of actin stress fibers, retraction of filopodia-like cellular protrusions, and formation or augmentation of F-actin reservoirs. Cellular protrusions in all directions could retract (Figure 1A, label 2). Stress fibers in all directions could depolymerize (Figure 2, and movie S5). F-actin reservoirs (Figure 1A, label 3) formed or grew in, often near retracting cellular protrusions or depolymerizing stress fibers.

Reinforcement responses involved polymerization and thickening of actin stress fibers (Figure 1B, label 5, and Figure 2), extension of cellular protrusions (Figure 1B, label 4), and decreases in the sizes and numbers of F-actin reservoirs (Figure 1B, label 6), often near the site of the extension of cellular protrusions. Actin reservoirs appeared to serve as reservoirs feeding growth of stress fibers (e.g. movie S4, in which a nearby actin reservoir appears to feed growth of the stress fiber of label 5). Extension of cellular protrusions and growth of stress fibers occurred predominantly in the direction of applied stretch.

Monitoring the same transfected cell at different degrees of stretch showed qualitatively identical classes of cell responses, although the probabilities of responses by cellular processes (extension, no response, and retraction) varied with mechanical stretch (*χ* ^2^ test of the independence of treatment and control, *χ* ^2^ = 7.33, P(>*χ* ^2^) = 0.03). Although cells in the control group (no mechanical stretch) changed over 30 minutes of observation, all three classes of response for filopodia-like protrusions were equally likely in the control group (Figure 1D). Cellular protrusions responded actively to uniaxial ETC stretch, with increased stretch reducing the likelihood of reinforcement responses (Figure 1D). Several cells also constricted slightly following stretch (Figure 2A). Retraction of cellular processes occurred less frequently following stretch, with no retraction responses observed following 5% stretch; however, these observations were not significant statistically.

### Stress fibers form in response to stretch

Stress fiber growth was observed following stretch at both strain levels. We examined results in Figure 1D using Fisher's exact test to examine any effect of the level of stretch on formation of stress fibers. The statistical results revealed the tendency for stress fibers to grow following stretch was independent of the degree of stretch level over the range tested (*p* = 0.25).

The time course of stress fiber reinforcement was particularly clear in a spindle cell with stress fibers that were well structured before stretch (Figure 2A). Such cells were found most commonly on the inner and outer extremities of the ring-like ETCs, with their natural axes aligned circumferentially on the ETC. Remodeling propagated from one apex of the cell (Figure 2B). In stellate cells that did not exhibit cytoskeletal alignment before stretching, post-stretch cytoskeletal rearrangement tended to direct stress fibers into the direction of ETC stretch (movie S6 and S7). Stretch-induced stress fiber formation was not observed following inhibition of rho kinase in stretched ETCs treated with 10 µM Y-27632 (Figures S5 and S6, supporting material), or following Ca^2+^ chelation (data not shown).

### Cytoskeletal responses follow distinct temporal sequences

**Figure 3:**
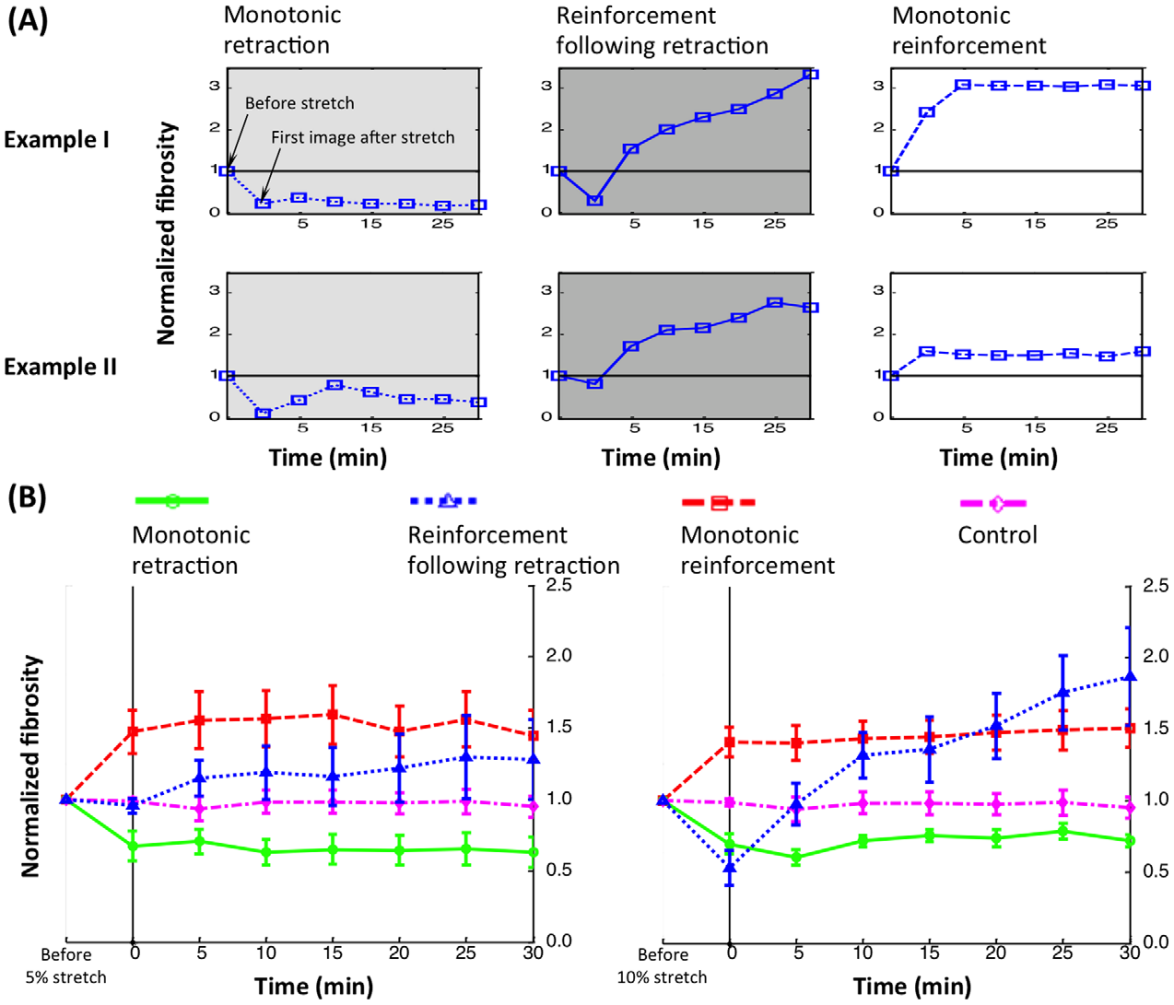
Three types of cytoskeletal response were observed following stretch (A, with two examples shown). The first time point imaged in each case was approximately two minutes after stretch, allowing sufficient time for stretch-induced cytoskeletal depolymerization to occur. The normalized fibrosity metric measures changes in the total length of features in an image that are within the thickness range of stress fibers (“fibrosity”). These three classes of response were significantly different relative to an unstretched control (B). Each curve represents the average of at least three cells that were imaged at both 5% and 10% stretch; error bars are standard error.

Three temporal patterns of cytoskeletal response were evident (Figure 3A). Although the responses were of different magnitude, the qualitative trends were clear. *Monotonic reinforcement* involved an increase in fibrosity following stretch, including extension of filopodia and growth of stress fibers, to a sustained level. This final sustained level was higher than that of unstretched controls (Figure 3B). Following 10% stretch, fibrosity increased on average ∼50%. *Monotonic retraction* involved a shift towards more F-actin reservoirs and fewer and smaller stress fibers and cellular protrusions, with no recovery of the latter (Figure 3A). Fibrosity dropped ∼20–40% and varied little, although drops after 5–10 minutes were sometimes evident (Figure 3A). *Reinforcement following retraction* involved a prompt fibrosity decrease followed by a gradual increase to a peak, sustained level near that of cells exhibiting monotonic reinforcement.

The class of temporal response did not depend upon cell shape or orientation, and multiple responses could be observed from a single cell when loaded repeatedly. For example, the spindle cell of Figure 2 exhibited monotonic retraction following 5% stretch, and reinforcement following retraction following 10% stretch.

Passive cell area changes following stretch were negligible, as expected for ETCs with Poisson's ratio of 1 (see Discussion S1 and Figure S1 in the supporting material). Area and perimeter changes were small compared to fibrosity changes, indicating that these contributed little to fibrosity measurements relative to changes to stress fibers.

Active contraction stresses were estimated by treating stretched ETCs with Y-27632 after a time interval sufficient to allow for viscoelastic relaxation. Reduction in isometric force following Y-27632 treatment indicated the degree of active cellular contraction (Figure S7, supporting material). The magnitude of active cellular contraction was independent of the magnitude mechanical stretch in ETCs, consistent with earlier observations [21].

### The cytoskeletal responses observed are consistent with a first order mechanical model

**Figure 4:**
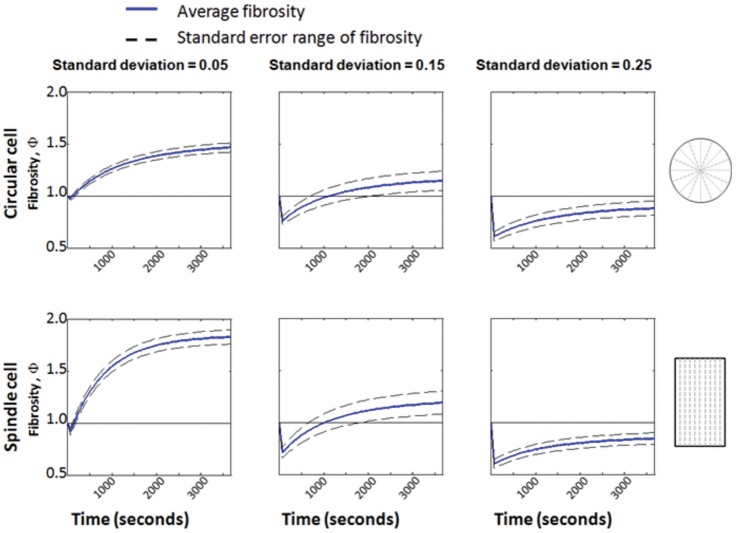
The three classes of dynamic cellular response could be captured by the model for all cell shapes considered, including circular (top row) and spindle-shaped (bottom row) cells. Populations of stress fibers were studied with the same mean pre-stretch, but with three different standard deviations. Monotonic reinforcement (first column), reinforcement followed by retraction (second column), and monotonic retraction (third column) were all predicted, depending upon the variation in pre-stretch within the fibers. Cells in which many stress fibers ruptured in tension underwent monotonic retraction. Cells in which no stress fibers ruptured in tension underwent monotonic reinforcement. All other cellular responses lay between these two extremes, and involved reinforcement following retraction.

Idealized “cells” (cartoons, Figure 4) studied through a mechanical model responded in a way that was consistent with experimental observations, including (1) retraction responses possible in all directions, (2) reinforcement occurring predominantly in the direction of stretch, and (3) the three classes of temporal response. As discussed in the next section, the features of the model responsible for this were the random distribution of stress fiber pre-stretches, which could result in stress fibers near both the tensile and compressive limits of the allowable range of pre-stretches, and the fact that the Poisson ratio of the ETCs was nearly 1 (Figure S1, supporting material), which resulted in large compressive strains transverse to the stretch direction.

**Figure 5:**
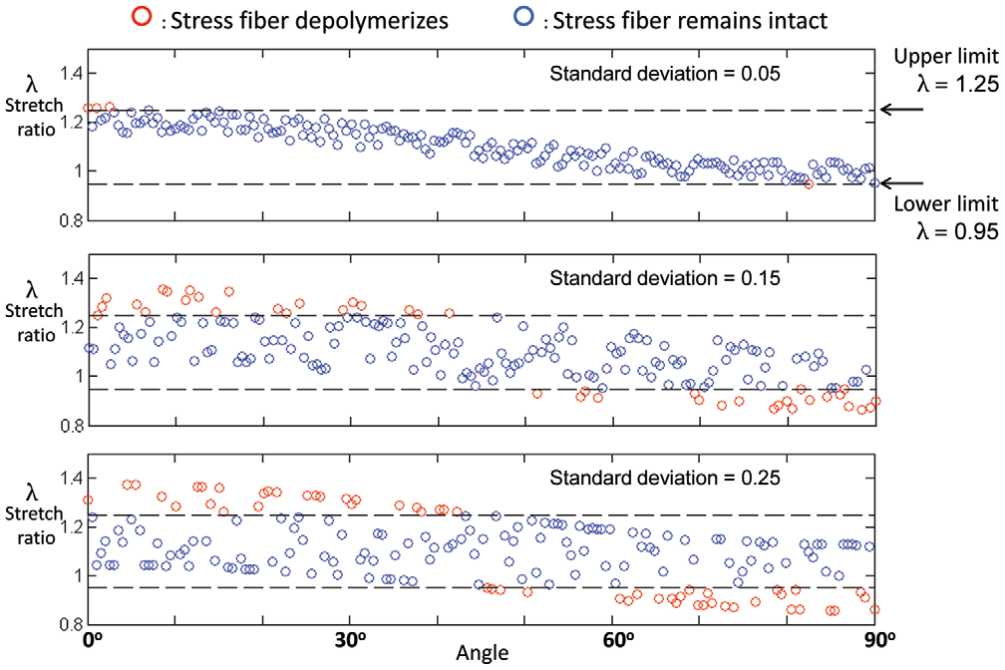
Model predictions of whether or not sectors of stress fibers in a circular cell would depolymerize following ETC stretch. Symbols represent the degree of stretch in stress fibers in individual sectors that would exist after the application of stretch to the ETC if depolymerization of stress fibers did not occur. Depolymerization does occur when stretch in a sector lies outside of a prescribed range, and the red symbols represent sectors that were predicted to depolymerize. For very low variation of stress fiber pre-stretch (top panel), only sectors nearly parallel (0°) to or perpendicular (90°) to the direction of ETC stretch were predicted to depolymerize. For higher variance in pre-stretch values (bottom two panels), the direction-dependence of depolymerization became weaker.

**Figure 6:**
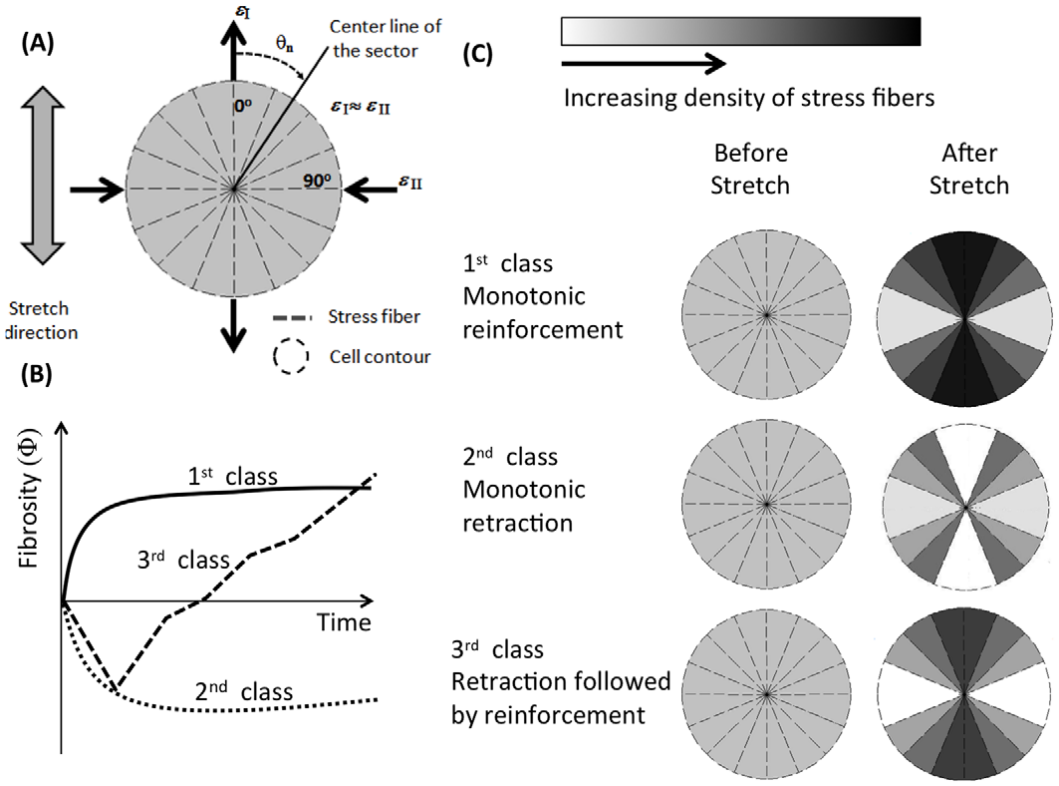
Data supported a hypothesis that the global cellular phenomena observed were triggered by local physical cues. (A) A mathematical model was developed to test this on idealized cells, as described in detail in the supplemental methods section. Shown here are cartoons of idealized circular cells containing a population stress fibers that changes over time according to our model. Cells were divided into sectors, and stress fibers in each sector were assigned a level of pre-stretch. Sectors that were stretched or compressed outside of the allowable range depolymerized (white); those that did not depolymerize and that exerted sufficient stress on their adhesions became denser in stress fibers (darker). (B) All three types of temporal response were reproduced by the model. (C) Cells with lower variability in stress fiber pre-stretch levels tended to exhibit monotonic reinforcement and polarization, with sectors aligned with ETC stretch becoming denser with stress fibers. Those with higher variability tended to exhibit monotonic retraction or reinforcement following retraction.

For a circular model cell with radial stress fibers, depolymerization of stress fibers could occur in any direction, provided that the statistical variance in the pre-stretch of fibers was sufficiently high. For a circular cell with very low variation of stress fiber pre-stretch, only sectors nearly parallel to or perpendicular to the direction of ETC stretch were likely to depolymerize following stretch (top panel of Figure 5). In Figure 5, symbols correspond to the degree of stretch that would exist following stretch of the ETC if stress fibers did *not* depolymerize. The roughly sinusoidal variation with respect to orientation is a result of the fact that Poisson's ratio is approximately 1 for these tissue constructs. As a consequence, a uniaxially loaded ETC produces compressive strains of the same order as tensile strains: a uniaxial ETC straining of amplitude *ε*_o_ in a direction *θ* = 0° imparted a strain *ε*_I_ *= ε*_o_coS3*θ* in a stress fiber oriented at angle *θ* (Figure 6A), yielding a transverse compressive strain (cf. Equation (1) in Methods S1, supporting material).

The red symbols in Figure 5 represent sectors that would be predicted to depolymerize following ETC stretch. For a cell with relatively low standard deviation in the pre-stretch of stress fibers, ETC stretch caused depolymerization of stress fibers that were either close to the direction of stretch or approximately perpendicular to the direction of stretch (top panel, Figure 5). For increasing standard deviation of the pre-stretches in sectors (bottom two panels, Figure 5), an increasing fraction of stress fiber sectors could reach the depolymerization limits following ETC stretch, and the direction-dependence of depolymerization reduced. Thus, for cells that presented no natural axis prior to ETC stretch, a sufficiently large scatter in the prestretches within stress fibers could explain the experimental observation that retraction responses could occur in any direction.

Following stretch of a magnitude associated with our experiments, three classes of response were observed, modulated by the variance in stress fiber pre-stretch relative to 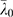 in both round cells and nominally spindle-shaped cells (Figure 4). Populations of stress fibers were studied with the same mean pre-stretch, but with three different standard deviations. Cells with little variance in stress fiber pre-stretch were likely to lose few sectors/compartments of stress fibers following ETC stretch, and tended to undergo monotonic reinforcement (Figure 4, left column). Intermediate levels of variance resulted in more sectors/compartments depolymerizing, but much regrowth (Figure 4, center column). This resulted in reinforcement following retraction. Sufficiently high variance in pre-stretch of stress fibers, or high initial pre-stretch of stress fibers, could result in a release of stress in the ECM surrounding the parts of the cell that undergo tension, and thereby lead to a monotonic retraction response (Figure 4, right column). Scatter was lower for the highest level of variation in fiber pre-stretch (right column) than for the intermediate level (center column), possibly because with the higher variation more fibers began the virtual experiment in a state that was near a critical state for depolymerization. If, following ETC stretch, the majority of stress fibers that undergo tensile straining depolymerize, the loss of stress at the adhesion sites slows or eliminates the reinforcement responses. In all cases, as in our experimental observations, reinforcement responses occurred predominantly in the direction of applied stretch.

### Stretch-induced stress fiber formation requires the Ca^2+^ and rho pathways

As expected from observations in 2D [39], stretch-induced stress fiber formation was not noticeable following inhibition of rho kinase in stretched ETCs treated with 10 µM Y-27632 (Figures S5 and S6, supporting material). In the test shown, fibrosity did not change relative to control following 5% ETC stretch. However, significant morphological change could occur in the absence of the rho kinase pathway, and the fibrosity of the cell shown in Figure S5 (supporting material) increased slightly following 10% stretch as the cell contracted actively from its adhesions, increasing the total length of filopodia. Consistent with results from the literature [19], stress fibers failed to regenerate following stretch in stretched ETCs in which extracellular Ca^2+^ was depleted using EGTA and intracellular Ca^2+^ was depleted using thapsigargin and BAPTA AM (results not shown).

## Discussion

### Contractile fibroblasts exhibit specific responses to mechanical stretch

Cells in 3D ETCs exhibited responses to stretch that were diverse, but that followed a unifying set of underlying principles, with local physical cues signaling cell-wide changes. Cellular responses included retraction and then, in many cases, reinforcement leading to alignment towards the direction of a stretch over the course of a few minutes. Retraction responses could occur for stress fibers and cellular protrusions oriented in any direction, while reinforcement occurred predominantly in the direction of applied ETC stretch. No retraction responses were observed following 5% stretch, suggesting a critical strain threshold for retraction responses, and also suggesting that mild stretch might stabilize stress fibers and cellular protrusions. Dynamic cellular responses to mechanical stretch followed three general classes of behavior. Monotonic reinforcement involved extension of stress fibers and cellular protrusions into the direction of ETC stretch. Monotonic retraction involved depolymerization of stress fibers and retraction of cellular processes. Retraction followed by reinforcement was observed frequently, but the opposite never occurred.

These specific responses could be predicted from a simple set of mechanical rules, as described below, but were also sensitive to rho kinase and Ca^2+^. Stress fiber formation required the rho kinase pathway and was Ca^2+^-dependent, suggesting that both the myosin regulatory light chain kinase pathway and rho kinase pathway are essential for stress fiber formation in ETCs (cf. [40]). Filopodium-like processes could retract even following treatment with Y-27632, which inhibits ROCK and therefore interferes with the RhoA/ROCK pathway and stress fiber contraction.

The dynamics of filopodium-like processes were affected by mechanical stretch. Sufficient mechanical stretch increased the likelihood of extension of these processes, but reduced the likelihood of retraction. This is consistent with the principles of the model, with stress-induced process extension more probable when processes are not close to the threshold for detachment.

### Dynamic cell-level responses derive from local physical cues

The mathematical model, applying three governing principles to stress fiber behavior, could predict a broad range of our experimental observations. As discussed later, the first two principles derived from the observations of others for cells cultured on 2D substrata. The first principle was that stress fibers collapse when their stretch is outside of a preferred range: both tensile and compressive strains can depolymerize stress fibers through rupture or buckling, respectively. Second, stress near an attachment between cell and ECM governed reinforcement. Note that for the ETCs, ECM stiffness also increases with stress, meaning that we could not with confidence distinguish between effects of stress and stiffening in this system. The third principle derived from our observations of clusters of F-actin within cells: F-actin reservoirs provide a mechanism by which cells can reinforce rapidly. F-actin clusters were included implicitly in the mathematical model by the absence of a conservation law for the quantity of actin in stress fibers.

These principles explain the observations in this article in the following ways. Retraction responses could occur for stress fibers in any orientation because of a combination of two factors. First, Poisson contraction was such that a tensile strain caused a transverse compressive strain of nearly equal magnitude, and second, because a distribution in the pre-stretch of stress fibers develops over a cell's mechanical loading history [41], stress fibers near a critical point for depolymerization in tension or compression can exist in all orientations. Although, as shown in Figure 5, a region exists at ±45° to the loading direction in which strains due to ETC loading are small, an increased likelihood of stress fibers in these orientations surviving a mechanical stretch was not observed in experiments. This effect was not evident in experiments, but cannot be ruled out because the sample size of cells containing clear features oriented at ±45° to the loading direction was very small.

The time course of reinforcement responses following stretch were consistent with a law of growth rates increasing with post-stretch stress in the ECM near an adhesion site (Figure 6B). The model predicted that three classes of temporal response are possible, regardless of the shape of the cells. In the context of the model, the choice amongst the three possible temporal responses was governed by the likelihood that stress fibers would remain intact in the direction of stretch. This was predicted by the degree of variation in the pre-stretch of cells.

This was true for the two extremes that were studied: cells with no natural axis, and cells with a highly defined natural axis. In both cases, the variation in pre-stretch in stress fibers modulated between the three types of temporal response observed. Monotonic reinforcement was predicted for cells with little variation in pre-stretch, with few stress fibers depolymerizing and many adhesion sites triggering stress fiber growth. Monotonic retraction was predicted for some cases of high variation of pre-stretch when a sufficient fraction of stress fibers ruptured in tension so as to drop the stress level at adhesion sites below that needed for stress fiber growth. Retraction followed by reinforcement was predicted for intermediate variation, but reinforcement followed by retraction never occurred in the model. This is explained by a separation of time scales for retraction and reinforcement.

The model also predicted polarization of cells into the direction of ETC stretch. Reinforcement occurred predominantly in the direction of stretch in the model, where stresses at adhesion sites rose to sufficiently high levels. Stress fibers aligned with the stretch direction that survived the ETC stretch were central to both the reinforcement and polarization responses, as indicated by the sectors near *θ* = 0° in Figure 6C. These sectors become dense with stress fibers (darker sectors) while the ETC is held isometrically unless the ETC stretch depolymerizes them (white sectors).

Cells in ETCs showed specific responses to mechanical stimulation that could be predicted through a few governing principles as implemented in a simple model. We note that factors other than those modeled are certainly important in cellular responses. Active contraction of cells is sometimes initiated in response to stress, and fibrosity can increase or decrease as cells pull back against adhesion sites. The magnitude of active cellular contraction we measured at the level of an ETC was independent of the magnitude of mechanical stretch in the ETC, consistent with earlier observations [21]. We emphasize that the model aimed to identify possible driving forces for the major trends observed. In one case out of the hundreds of cells observed experimentally, a cell retracted into a ball, then elongated into a nominally spindle-shaped cell. This latter case cannot be modeled using the approach presented, and is mentioned to highlight that the scope of this model is limited to a specific set of phenomena that seem to govern the vast majority of our observations. However, the governing principles identified are predictive of the major trends, and might have broader implications for development and remodeling of tissues.

### Cytoskeletal dynamics in 3D differ from those observed in 2D

Many of the behaviors we observed in 3D ETCs have direct analogs in observations from cells cultured on 2D substrata. Post-stretch retraction responses were consistent with the Fredberg model [17] of cytoskeletal fluidization induced exclusively through mechanical means in 2D culture. Consistent with their observations, the time scale for stress fiber depolymerization was too short to be resolved by our methods. The dependence of cellular behavior on the distribution of stress fiber pre-strains, as applied by the model, was consistent with the models of Kaunas for cells loaded cyclically on a 2D substratum (e.g. [26]–[30]). Although data are not available for the allowable stretch range of stress fibers in cells within ETCs, applying the range reported by the Kaunas group to our models allowed us to replicate our experiment results qualitatively. The realignment of cells and extension of filopodia into a direction of sustained stretch is also consistent with this work, as well as with a wealth of data and models predicting polarization of endothelial cells adherent to flexible 2D substrata subjected to very low frequency stretch [33]–[37]. The concept of stress-driven modulation of actin stress fiber density is consistent with the 2D Deshpande model [32], and the concept of stress fibers aligning due to elastic interactions with the ECM is consistent with the recent work of Safran and collaborators [42], [43].

However, several differences exist. Contrary to observations of cells in 2D, retraction and reinforcement responses were both observed during isometric stretches, with retraction responses possible in all directions. In 2D F-actin depolymerization is observed predominantly in unloading of the actin cytoskeleton following stretch [44]. F-actin depolymerization was observed here in 3D both following sustained stretch, and also in compressed stress fibers.

### Organization of F-actin in ETCs differed from that observed in 2D

An important feature is that F-actin reservoirs appeared to play a role in cytoskeletal dynamics. Reservoirs tended to disappear when stress fibers formed, and to appear when stress fibers disappeared. Reservoirs were also associated with the dynamics of filopodia-like cellular protrusions. While F-actin dynamics in cells on a 2D substratum is limited by F-actin availability [41], these reservoirs appear to allow a more rapid response for cells in 3D, and thus play a role in altering gross cell shape towards spindles aligned with the direction of stretch. However, many open questions persist about these structures and their function. Further study of their kinetics, relation to other cytoskeletal elements, and possible roles in the range of cellular functions is warranted.

An additional difference was that cells in 3D exhibited a single natural axis defined by stretch, in contrast to cells in 2D with an additional axis defined by the normal to the culture dish. Ventral stress fibers, dorsal stress fibers and transverse arcs [41] were not observed.

Lamellipodia were absent in all cells tested (*n*>100), while F-actin reservoirs were evident in all cells. Nevertheless, observations of 2D lamellipodia dynamics may shed some light on the role and function of the F-actin reservoirs. Lamellipodia are relatively stiff networks of F-actin [45], and the F-actin in these networks exhibits retraction to rounded masses in neurons cultured in 2D and treated with cytochalasin B [46], possibly due to contractile tension in the fibers [47]. In 3D ETCs, F-actin reservoirs appeared at times when other cytoskeletal features such as stress fibers and filopodium-like cellular processes disappeared, and disappeared when these other features reappeared, again suggesting that they might serve as reservoirs, or might in fact contain fragments of stress fibers that have retracted, as discussed below.

Stress fibers typically display a periodic α-actinin–myosin II pattern and therefore are generally thought to resemble the sarcomeric actin filament structures of muscle cells. In our studies the repeated patterns were not shown clearly in 3D culture, despite the fact that the cells showed this repeated pattern when cultured in 2D (Figure S3, supporting material). This is consistent with other observations of stress fibers in 3D gels [48]. *α*-actinin was associated with patches like those we term F-actin reservoirs, suggesting that these might contain segments of stress fibers. The reservoirs themselves were likely not artifacts of transfection, as analogous structures appear rhodamine phalloidin stained fibroblasts that were not transfected. These large reservoirs might be related to disordered actin clusters observed at the nanoscale (e.g. [49]) that can coalesce into stress fibers [50].

### Concluding remarks

Cells in ETCs showed staged retraction and reinforcement in response to mechanical stimulation. Retraction responses occurred for all orientations of stress fibers and cellular protrusions relative to the stretch direction, while reinforcement responses, including extension of cellular processes and stress fiber formation, occurred predominantly in the stretch direction. These responses could be understood through a few governing principles that highlight the importance of mechanical straining in the behavior of a cell. Specifically, the range of observed responses could be explained through variability in the strain history of stress fibers, through mechanical limits on the stretch allowable within a stress fiber, and through responsiveness of stress fibers to the mechanical environment at their attachment to the extra-cellular matrix. The governing principles identified were predictive of the major trends observed, and might have broader implications for development and remodeling of tissues.

## Supporting Information

Figure S1 **Strain fields surrounding cells were estimated by tracking and analyzing displacements of fluorescent beads using standard techniques that reviewed elsewhere** [**52**] **.** The Poisson ratio was approximately 1, indicating significant ECM anisotropy. Shown here are local strains as a function of the nominal strains on the ETC. Standard methods were used to estimate the strain fields within the planar flanks of rubber band-like ETCs after the mechanical stretch. Compared to 10% nominal ETC stretch, 30% nominal ETC stretch produced greater elongation parallel to the stretch direction and greater Poisson contraction perpendicular to the stretch direction. The relationship between nominal ETC stretch and stretch within the planar flanks of the ETCs was nonlinear. However, over the range tested the effective Poisson ratio was ∼1, which is within the thermodynamic bounds for a transversely isotropic material.
(TIF)

Figure S2 S**ymbolic representation of the simplified mechanical model.** The procedure involved initialization of the model, calculation of stretch ratio of each sector of stress fibers, decision-making to determine whether depolymerization of stress fibers occurred, and evaluation of reinforcement rates (gray box).
(TIFF)

Figure S3 **Stress fibers and actin reservoirs contain organized** ***α*** **-actinin.** Immunostaining of fixed cells showed that clumps termed “F-actin reservoirs” contained both F-actin and *α*-actinin. F-actin reservoirs appeared in all transfected cells (e.g., Figure 1 from the main text). Here, analogous clumps appeared in fixed, non-transfected cells that were stained with rhodamine phalloidin (white stain, top right panel). This indicates that F-actin reservoirs were not an artifact of transfection and that they contain F-actin. F-actin reservoirs did not appear in identical cells cultured on a 2D substratum that were fixed and stained with rhodamine phalloidin (white stain, top left panel). Stress fibers and actin reservoirs observed in cells within ETCs displayed organized but poorly striated distributions of *α*-actinin (red stain, lower panel). Analogous clumps appeared. The image on the right was obtained following preconditioning and a mechanical stretch of 10%; ETCs were fixed in their stretched state using 4% paraformaldehyde, then stained with both primary and secondary antibodies using standard procedures to reveal *α*-actinin. Note that *α*-actinin is much less organized in non-muscle cell stress fibers than muscle fibers (e.g. [48]). This is evident from *α*-actinin staining of identical, fixed cells that were cultured on a 2D substratum (red stain, lower left panel).
(TIF)

Figure S4 **ETCs responded viscoelastically and actively to mechanical stretch.** Following mechanical stretch, the isometric force needed to sustain a specimen at prescribed length increased above its baseline level, then decreased to slightly above the baseline pre-stretch value over the course of approximately 30 minutes as the ECM and cells relaxed viscoelastically and as the cells remodeled actively. Force data shown here was recorded while an ETC was subjected to a mechanical stretch of 30%. The force relaxation curve is typical. The high frequency oscillations were due to noise. Lower frequency oscillations were evident after data were filtered using a moving window average. However, low frequency oscillations also appeared in filtered force relaxation curves for ETCs treated with deoxycholate, so no conclusions can be drawn from these data about contributions of active cellular contractions.
(TIF)

Figure S5 **Cell morphology and mechanics were assayed in ETCs that were treated with 10 µM Y-27632, then stretched by 10% and 30% (resulting in nominal cell stretches of 5% and 10%) and held isometrically.** Results indicate that significant morphological changes can occur in the absence of the rho kinase pathway. In ETCs treated with Y-27632 then stretched by 30% and held isometrically, retraction and extension of filopodium-like cellular processes was evident, but formation of stress fibers was not observed. Blocking the rho kinase pathway using Y-27632 does not eliminate the ability of these cells to alter their morphology.
(TIF)

Figure S6 **Cell morphology and mechanics were assayed in ETCs that were treated with 10 µM Y-27632, then stretched by 10% and 30% (resulting in nominal cell stretches of 5% and 10%) and held isometrically.** Shown here is the time course of fibrosity during these experiments. The fibrosity was unaffected by stretch in these cells.
(TIF)

Figure S7
**Active contractile force associated with Y-27632 was independent of the degree to which the ETC was stretched.** Y-27632 was added to tissue constructs that had been stretched then held isometrically for a time interval to allow for viscoelastic relaxation to a nominally steady state force. The subsequent reduction in steady state force was associated with active cellular contraction, and was normalized by the approximate number of cells in the tissue construct. This force was approximately Δ*F*  = 110 nN, and was independent of stretch level. Shown here are effects of Y-27632 on force responses of (A) an ETC stretched 10% and (B) a different ETC first stretched 10%, and then stretched 30%; in both cases, the preconditioning and relaxation protocols decribed in the main text were followed. The reduction in active contractile force (and, as described below, the active contractile stress) associated with the addition of Y-27632 was independent of the level of stretch. These observations support those of [21] that cellular contractile forces are independent of the degree to which cells are stretched in a 3D ETC. The average active stress was estimated from this as 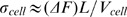, where *L = *0.007 m is the approximate spacing between loading bars (Figure 1C), and *V_cell_* ∼ 10^−12^ m^3^. This yields a coarse estimate on the order of 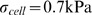, close to values reported for non-transfected cells [53]. The effect of stretch on this estimate is small.(TIF)

Methods S1 **A first order model of a cell as a assemblage of stress fibers was studied to assess the hypothesis that responses to passive, local, physical cues could combine to produce the global cellular remodeling sequences observed in our experiments.** For simplicity, we focused on stress fibers in the illustrations in this section. However, the extension to cellular processes is straightforward. The flowchart of the simplified model, specialized to this case, is illustrated in Figure S2 (supporting material). The inputs to the model were a cell shape, a population of stress fibers with prescribed orientations and levels of mechanical pre-stretch, and a local strain field resulting from mechanical stretch of an ETC. Outputs of the model were predicted behavior of stress fibers over time (polymerization and depolymerization) and an estimate of the fibrosity measure that would be observed experimentally. Two basic principles governed stress fiber dynamics in the model. First was the widely-reported observation that stress fibers depolymerize with too great or with insufficient stretch (e.g. [26], [27], [31]–[34], [36]). Specifically, the work of the Kaunas group has quantified a range of stretch ratios over which a stress fiber can exist for endothelial cells, independent of the shape or size of a cell [26], [27], [39]. Stress fibers contract over time as a function of the mechanical environment of a cell, so that their “prestretch” increases. The second was a hypothesis that peak stress at an adhesion site drove stress fiber polymerization towards a maximum allowable density over the time window modeled. An underlying principle was that F-actin reservoirs provided a store of F-actin or possibly stress fiber fragments that could be recruited rapidly to form new stress fibers or cellular protrusions.
(PDF)

Discussion S1 **A simple example was studied to estimate the degree of passive changes to cell area and perimeter in response to stretch.** This model was predicated on the observation that contractile fibroblasts and ECM have approximately the same elastic moduli in ETCs under the conditions tested, in which ETCs contain a population of cells just above the percolation threshold [51]. In this case, the strain in the local ECM should approximately equal that in the cells.
(PDF)

Movie S1 **Stretch response of a spindle-shaped cell.** The cell was stretched in the vertical direction. The cell presented clear stress fibers prior to ETC stretch. Several of these stress fibers depolymerized following ETC stretch. Stress fibers grew over the course of 30 minutes as the ETC was held in a state of isometric stretch. Differences between subsequent images (Figure 2 in the main text) suggest a time course of cellular remodeling.
(AVI)

Movie S2 **Prior to mechanical stretch, cells extended and retracted filopodium-like processes in all directions.** The dynamics of these processes was associated with the appearance and disappearance of F-actin reservoirs.
(AVI)

Movie S3 **Time course of the response of the cell in** **Figure 1A** **to mechanical stretch.** The cell was stretched in the vertical direction. Notable features include retraction of cellular processes and formation and enlargement of F-actin reservoirs.
(AVI)

Movie S4 **Time course of the response of the cell in** **Figure 1B** **to mechanical stretch.** The cell was stretched in the vertical direction. Notable features include formation of stress fibers and extension of cellular processes into the direction of mechanical stretch. These occurred through an apparent trade-off with F-actin reservoirs.
(AVI)

Movie S5 **Stress fibers and cellular protrusions in any direction could undergo retraction responses.** In the upper left branch of the cell shown here, stress fibers that are nearly perpendicular to the stretch direction (stretch direction: vertical) depolymerize following stretch. These images were deconvoluted using standard techniques and a theoretical point spread function to highlight stress fibers.
(AVI)

Movie S6 **Cells tended to retract processes perpendicular to the stretch direction (the stretch direction was vertical) and extend stress fibers and processes into the stretch direction.** The cell shown is responding to a 5% stretch. Following 10% stretch (Movie 7), the shape transition continued towards a spindle-like morphology with a natural axis aligned with the direction of stretch.
(AVI)

Movie S7 **Cells tended to retract processes perpendicular to the stretch direction (the stretch direction was vertical) and extend stress fibers and processes into the stretch direction.** The cell shown is responding to a 10% stretch; the response to 5% stretch is shown in Movie 6. Here, the cell continues a shape transition towards a spindle-like morphology with a natural axis aligned with the direction of stretch.
(MP4)
