# Supplementary figures and images for: Physically-Induced Cytoskeleton Remodeling of Cells in Three-Dimensional Culture
Source: PLoS One. 2012 Dec 27;7(12):e45512. doi: 10.1371/journal.pone.0045512 (PMC3531413; doi:10.1371/journal.pone.0045512)

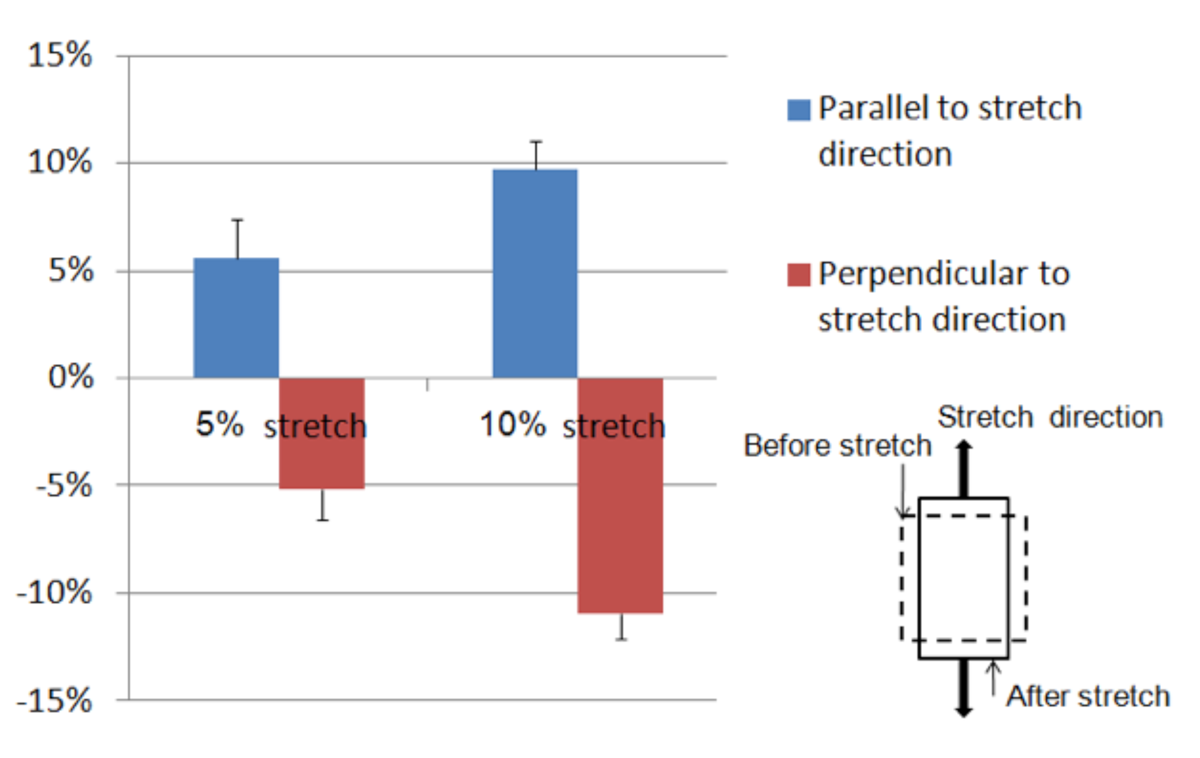

Supplement: Figure S1 — Strain fields surrounding cells were estimated by tracking and analyzing displacements of fluorescent beads using standard techniques that reviewed elsewhere [52] . The Poisson ratio was approximately 1, indicating significant ECM anisotropy. Shown here are local strains as a function of the nominal strains on the ETC. Standard methods were used to estimate the strain fields within the planar flanks of rubber band-like ETCs after the mechanical stretch. Compared to 10% nominal ETC stretch, 30% nominal ETC stretch produced greater elongation parallel to the stretch direction and greater Poisson contraction perpendicular to the stretch direction. The relationship between nominal ETC stretch and stretch within the planar flanks of the ETCs was nonlinear. However, over the range tested the effective Poisson ratio was ∼1, which is within the thermodynamic bounds for a transversely isotropic material. (TIF) [file pone.0045512.s001.tif]

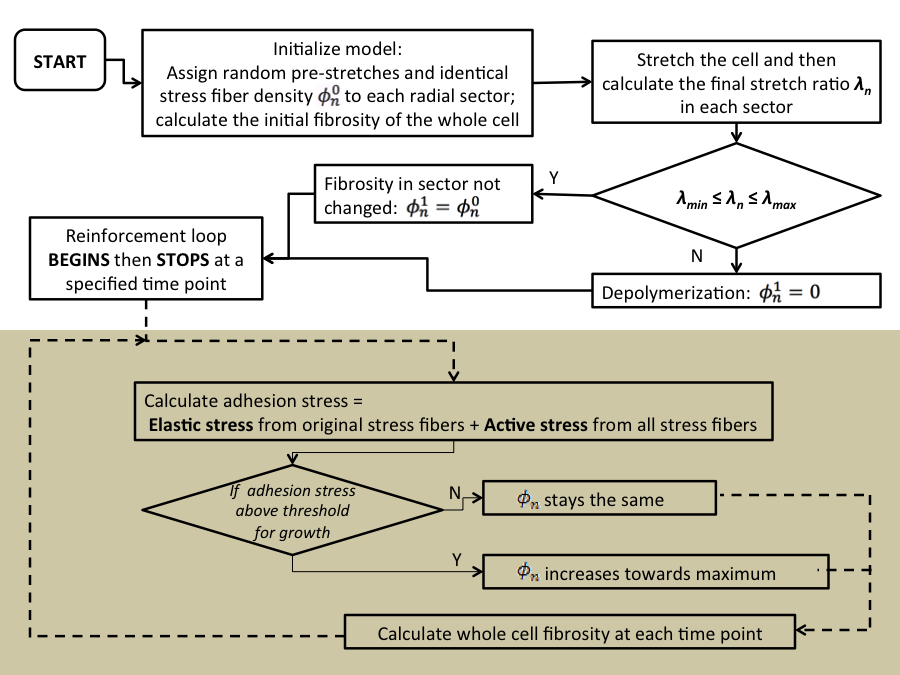

Supplement: Figure S2 — Symbolic representation of the simplified mechanical model. The procedure involved initialization of the model, calculation of stretch ratio of each sector of stress fibers, decision-making to determine whether depolymerization of stress fibers occurred, and evaluation of reinforcement rates (gray box). (TIFF) [file pone.0045512.s002.tif]

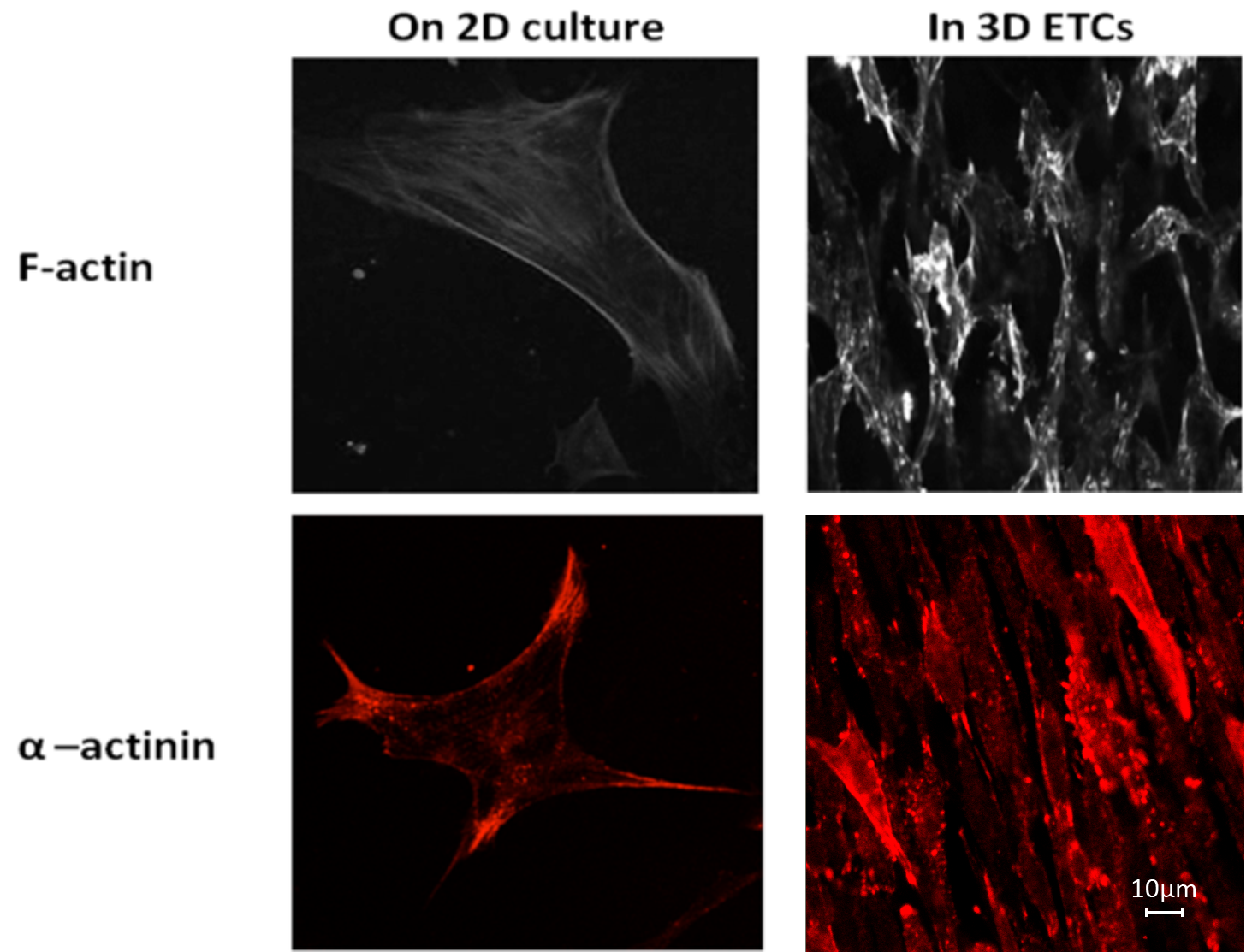

Supplement: Figure S3 — Stress fibers and actin reservoirs contain organized α -actinin. Immunostaining of fixed cells showed that clumps termed “F-actin reservoirs” contained both F-actin and α-actinin. F-actin reservoirs appeared in all transfected cells (e.g., Figure 1 from the main text). Here, analogous clumps appeared in fixed, non-transfected cells that were stained with rhodamine phalloidin (white stain, top right panel). This indicates that F-actin reservoirs were not an artifact of transfection and that they contain F-actin. F-actin reservoirs did not appear in identical cells cultured on a 2D substratum that were fixed and stained with rhodamine phalloidin (white stain, top left panel). Stress fibers and actin reservoirs observed in cells within ETCs displayed organized but poorly striated distributions of α-actinin (red stain, lower panel). Analogous clumps appeared. The image on the right was obtained following preconditioning and a mechanical stretch of 10%; ETCs were fixed in their stretched state using 4% paraformaldehyde, then stained with both primary and secondary antibodies using standard procedures to reveal α-actinin. Note that α-actinin is much less organized in non-muscle cell stress fibers than muscle fibers (e.g. [48]). This is evident from α-actinin staining of identical, fixed cells that were cultured on a 2D substratum (red stain, lower left panel). (TIF) [file pone.0045512.s003.tif]

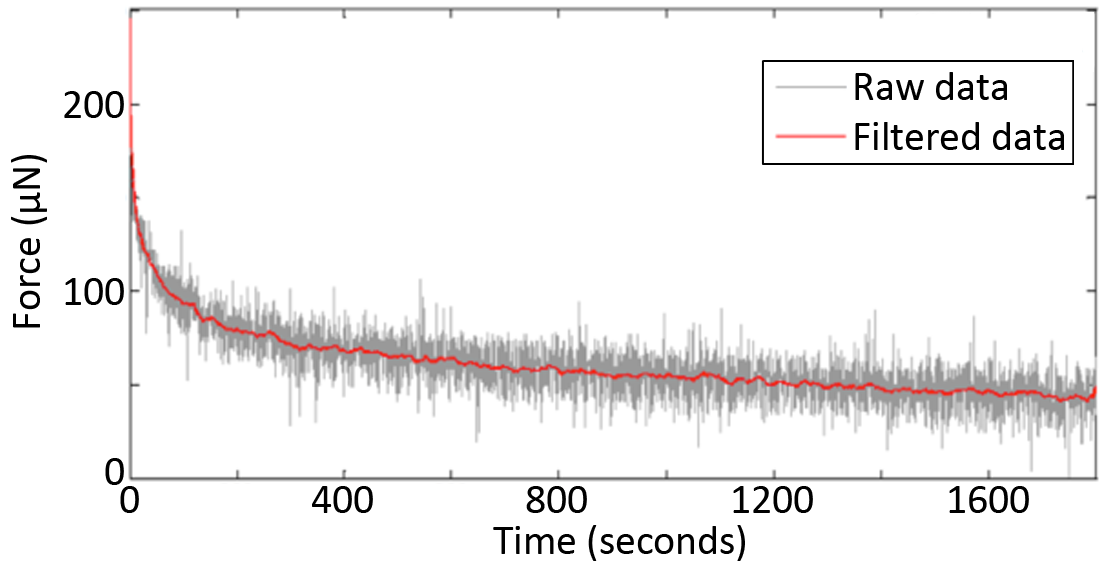

Supplement: Figure S4 — ETCs responded viscoelastically and actively to mechanical stretch. Following mechanical stretch, the isometric force needed to sustain a specimen at prescribed length increased above its baseline level, then decreased to slightly above the baseline pre-stretch value over the course of approximately 30 minutes as the ECM and cells relaxed viscoelastically and as the cells remodeled actively. Force data shown here was recorded while an ETC was subjected to a mechanical stretch of 30%. The force relaxation curve is typical. The high frequency oscillations were due to noise. Lower frequency oscillations were evident after data were filtered using a moving window average. However, low frequency oscillations also appeared in filtered force relaxation curves for ETCs treated with deoxycholate, so no conclusions can be drawn from these data about contributions of active cellular contractions. (TIF) [file pone.0045512.s004.tif]

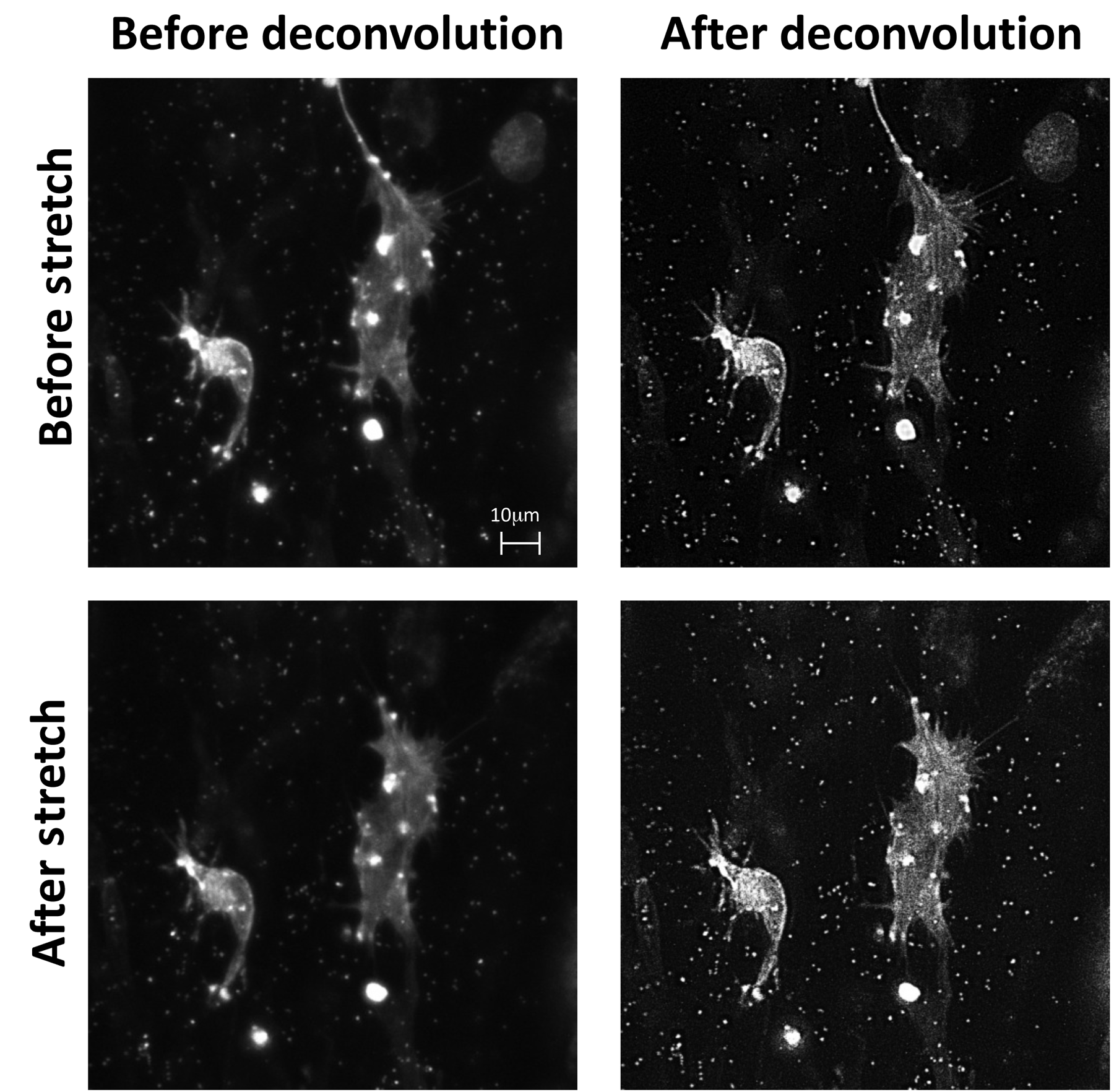

Supplement: Figure S5 — Cell morphology and mechanics were assayed in ETCs that were treated with 10 µM Y-27632, then stretched by 10% and 30% (resulting in nominal cell stretches of 5% and 10%) and held isometrically. Results indicate that significant morphological changes can occur in the absence of the rho kinase pathway. In ETCs treated with Y-27632 then stretched by 30% and held isometrically, retraction and extension of filopodium-like cellular processes was evident, but formation of stress fibers was not observed. Blocking the rho kinase pathway using Y-27632 does not eliminate the ability of these cells to alter their morphology. (TIF) [file pone.0045512.s005.tif]

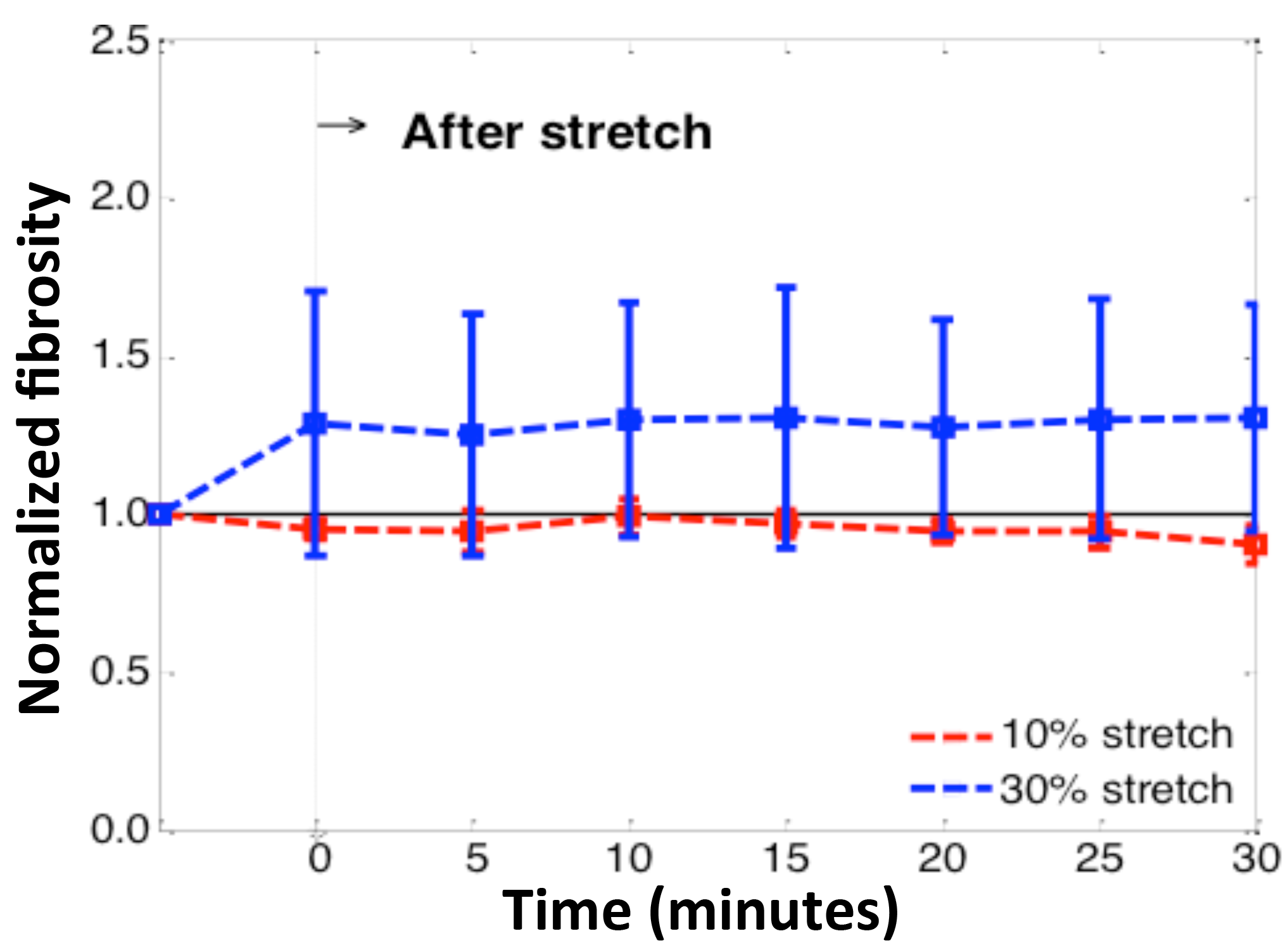

Supplement: Figure S6 — Cell morphology and mechanics were assayed in ETCs that were treated with 10 µM Y-27632, then stretched by 10% and 30% (resulting in nominal cell stretches of 5% and 10%) and held isometrically. Shown here is the time course of fibrosity during these experiments. The fibrosity was unaffected by stretch in these cells. (TIF) [file pone.0045512.s006.tif]

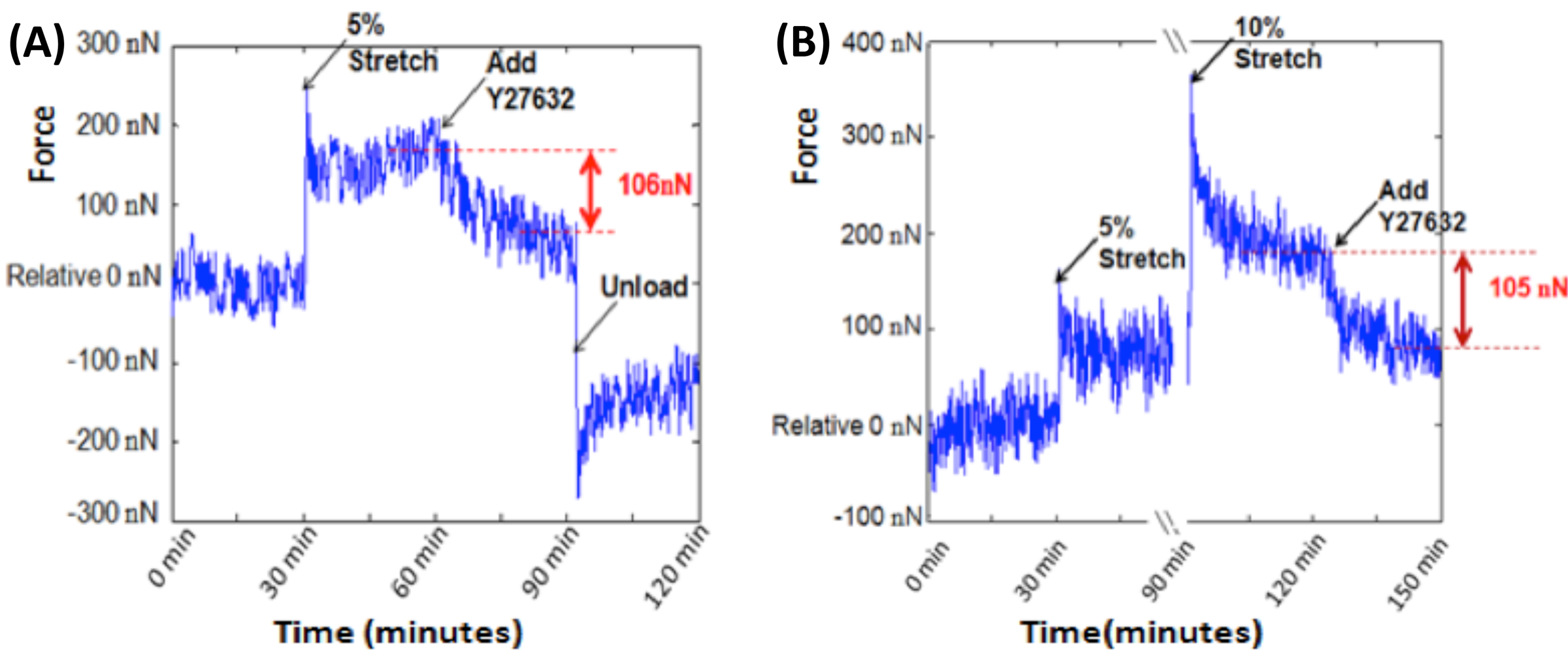

Supplement: Figure S7 — Active contractile force associated with Y-27632 was independent of the degree to which the ETC was stretched. Y-27632 was added to tissue constructs that had been stretched then held isometrically for a time interval to allow for viscoelastic relaxation to a nominally steady state force. The subsequent reduction in steady state force was associated with active cellular contraction, and was normalized by the approximate number of cells in the tissue construct. This force was approximately ΔF = 110 nN, and was independent of stretch level. Shown here are effects of Y-27632 on force responses of (A) an ETC stretched 10% and (B) a different ETC first stretched 10%, and then stretched 30%; in both cases, the preconditioning and relaxation protocols decribed in the main text were followed. The reduction in active contractile force (and, as described below, the active contractile stress) associated with the addition of Y-27632 was independent of the level of stretch. These observations support those of [21] that cellular contractile forces are independent of the degree to which cells are stretched in a 3D ETC. The average active stress was estimated from this as , where L = 0.007 m is the approximate spacing between loading bars (Figure 1C), and Vcell ∼ 10−12 m3. This yields a coarse estimate on the order of , close to values reported for non-transfected cells [53]. The effect of stretch on this estimate is small. (TIF) [file pone.0045512.s007.tif]
