# Supplementary material for: Physically-Induced Cytoskeleton Remodeling of Cells in Three-Dimensional Culture
Source: PLoS One. 2012 Dec 27;7(12):e45512. doi: 10.1371/journal.pone.0045512 (PMC3531413; doi:10.1371/journal.pone.0045512)
Supplement: Methods S1 — A first order model of a cell as a assemblage of stress fibers was studied to assess the hypothesis that responses to passive, local, physical cues could combine to produce the global cellular remodeling sequences observed in our experiments. For simplicity, we focused on stress fibers in the illustrations in this section. However, the extension to cellular processes is straightforward. The flowchart of the simplified model, specialized to this case, is illustrated in Figure S2 (supporting material). The inputs to the model were a cell shape, a population of stress fibers with prescribed orientations and levels of mechanical pre-stretch, and a local strain field resulting from mechanical stretch of an ETC. Outputs of the model were predicted behavior of stress fibers over time (polymerization and depolymerization) and an estimate of the fibrosity measure that would be observed experimentally. Two basic principles governed stress fiber dynamics in the model. First was the widely-reported observation that stress fibers depolymerize with too great or with insufficient stretch (e.g. [26], [27], [31]–[34], [36]). Specifically, the work of the Kaunas group has quantified a range of stretch ratios over which a stress fiber can exist for endothelial cells, independent of the shape or size of a cell [26], [27], [39]. Stress fibers contract over time as a function of the mechanical environment of a cell, so that their “prestretch” increases. The second was a hypothesis that peak stress at an adhesion site drove stress fiber polymerization towards a maximum allowable density over the time window modeled. An underlying principle was that F-actin reservoirs provided a store of F-actin or possibly stress fiber fragments that could be recruited rapidly to form new stress fibers or cellular protrusions. (PDF) [file pone.0045512.s008.pdf]

Supplemental methods: First order model of cell dynamics

Sheng-Lin Lee, et al., Physically-induced cytoskeleton remodeling of cells in three-dimensional culture.

A first order model of a cell as a assemblage of stress fibers was studied to assess the hypothesis that responses to passive, local, physical cues could combine to produce the global cellular remodeling sequences observed in our experiments. For simplicity, we focused on stress fibers in the illustrations in this section. However, the extension to cellular processes is straightforward. The flowchart of the simplified model, specialized to this case, is illustrated in Figure S2. The inputs to the model were a cell shape, a population of stress fibers with prescribed orientations and levels of mechanical pre-stretch, and a local strain field resulting from mechanical stretch of an ETC. Outputs of the model were predicted behavior of stress fibers over time (polymerization and depolymerization) and an estimate of the fibrosity measure that would be observed experimentally.

Two basic principles governed stress fiber dynamics in the model. First was the widely-reported observation that stress fibers depolymerize with too great or with insufficient stretch (e.g. [26,27,31-34,36]). Specifically, the work of the Kaunas group has quantified a range of stretch ratios over which a stress fiber can exist for endothelial cells, independent of the shape or size of a cell [26,27,39]. Stress fibers contract over time as a function of the mechanical environment of a cell, so that their "prestretch" increases. The second was a hypothesis that peak stress at an adhesion site drove stress fiber polymerization towards a maximum allowable density over the time window modeled. An underlying principle was that F-actin reservoirs provided a store of F-actin or possibly stress fiber fragments that could be recruited rapidly to form new stress fibers or cellular protrusions.

Models of cells

Two limiting cases of pre-stretch cell morphology were modeled. The first limiting case represented the extreme of spindle-shaped cells such as that of Figure 2 from the main text. The model was a rectangular cell with its natural axis aligned in the direction of stretch. The cell contained a population of stress fibers with a uniform spatial distribution. All stress fibers were aligned with the natural axis of the cell. The initial pre-stretches of the stress fibers were selected randomly from a normal distribution. This was implemented by dividing the cell width into N rectangular compartments of equal width and of the length of the cell, then assigning all stress fibers within each compartment n the same pre-stretch value.

The second limiting case represented the observation that, prior to stretch, ETCs contained cells, stress fibers and cellular protrusions that could be oriented in any direction. The model was a circular cell containing a population of radial stress fibers that were centered in circle, with orientations distributed uniformly over the circle (see Figure 6 from the main text). The initial pre-stretches of the fibers were again selected randomly from a normal distribution. To implement this, the semicircle was divided into N sectors, and the stress fibers within each sector n were assigned a random pre-stretch value, λ_n^o; the sectors were symmetrical radially. The strain field in this case was governed by the initial orientation angle θ_n from the direction of ETC stretch to the centerline of the segment (see Figure 6 from the main text).

Application of ETC stretch

A uniaxial stretch was applied, straining the cell and its local environment by an amount ε_I. For the case of a spindle-shaped cell, all compartments of stress fibers strained by this amount. For the case of a circular cell, the degree to which stress fibers stretched depended upon the orientation angle θ_n of the sector n. The effective Poisson's ratio observed in experiments was $\nu \approx 1$ (Figure S1), meaning that $\varepsilon_{II} \approx \varepsilon_I$ (Figure 6, main text). The final stretch in the nth sector was:

$$\lambda_n^f = \lambda_n^o(1 + \epsilon_I(\cos 2\theta_n)). \tag{1}$$

Model for stress fiber depolymerization

Stress fibers within each of the N compartments or sectors of a cell had a randomly assigned initial pre-stretch λ_n^o, where n = 1, 2, …, N represents the compartment or segment number. The random distribution had a mean of $\bar{\lambda}_o$ =1.1 [26,27] and a standard deviation that was varied parametrically. Stress fibers depolymerized for λ_n outside of the range $\lambda_{min} \leq \lambda_n \leq \lambda_{max}$, where λ_{min} = 0.95 and λ_{max} = 1.25 (a reasonable range based upon [26-30,39]). To ensure a representative distribution, stress fibers whose initial, randomly assigned pre-stretch values lay outside of the prescribed range were assigned the nearest extreme value within the allowable range.

Depolymerization of stress fibers upon stretch is rapid compared to reinforcement responses [16,17,19,27]. Since our experimental measurements could not capture deoplymerization dynamics following the rapid stretch, these were not modeled carefully and were instead taken to occur before the first post-stretch measurement was made.

The degree of polymerization within each stress fiber compartment or sector was tracked at each timepoint i with a normalized stress fiber density, ϕ_n^i, that relates to the "fibrosity" measure obtained through image analysis in our experiments. ϕ_n^i was initially uniform over all sectors with $\phi_n^o = 1$. At the end of the first time increment following stretch, ϕ_n^1 became zero if the stretch in compartment or sector n was outside the allowable range. Otherwise, it was sustained at $\phi_n^1 = 1$.

The specific measure that corresponds to our experimental measurements is a total "normalized fibrosity," Φ^i, at each timepoint i. This was set based upon what would be seen in a "virtual" image of the deformed model cell at each point in time considered. Φ^i was calculated by summing the fiber densities ϕ_n^i in each sector or compartment n at each time i and adjusting for the strain in that sector or compartment: $\Phi^i = \left(\sum_{n=1}^{N} \phi_n^i(1 + \epsilon_n)\right)/\left(\sum_{n=1}^{N} \phi_n^o\right)$. The correction of $(1 + \epsilon_n)$ is needed to account for distortion of the cell: the fibrosity measure tracks the total length of stress fibers in an image, which increases with strain. For the spindle cells, $\epsilon_n = \epsilon_I$ for all n; for circular sectors, $\epsilon_n = \epsilon_I(\cos 2\theta_n)$.

Model for stress fiber growth in response to stress (or stiffness) near an adhesion site

We modeled subsequent growth of stress fiber density as being driven by normal stress σ_n at adhesion sites in the nth sector or compartment:

$$\frac{d\phi_n}{dt} = \frac{(\phi_{max} - \phi_n)}{\tau_G} f(\sigma_n) \tag{2}$$

where τ_G is a time constant, ϕ_{max} is an upper limit on stress fiber density, and $f(\sigma_n)$ is a function that determines stress fiber growth rates as a function of σ_n. τ_G = 4500s represents the time constant for gradual reinforcement observed in [19]; ϕ_{max} was taken as 2 for illustrative purposes. The function $f(\sigma_n)$ was taken as a linear function of σ_n for σ_n above a threshold value for stress fiber growth, σ_g:

$$f(\sigma_n) = \begin{cases} \alpha\left(\dfrac{\sigma_n}{\sigma_g} - 1\right), & \sigma_n \geq \sigma_g \\ 0, & \sigma_n \leq \sigma_g \end{cases} \tag{3}$$

where α is a proportionality constant that was set to 1 in simulations. Although much more sophisticated models for stress fiber stresses and kinetics exist [27,32], the simple form in Equation (6) is sufficient to show the essence of how stress responses might underlie our observations.

The estimates of σ_n involved an elastic component s_n^{el} due to stretch of the stress fibers in a sector or compartment, and an active component s_n^{active} due to actomyosin contraction. The active component was estimated based upon the following three observations. Results in [21] suggest that the active contractile stress in contractile fibroblasts is independent of stretch, although debate exists. Results in [19] suggest that active stress scales linearly with fibrosity Φ^i and thus stress fiber density. Results in this article corroborate estimates from the literature of an average active cellular stress of $s_o \approx 0.5$ kPa; consistent with [19], the degree of activation of all stress fibers was held constant so that s_o did not vary with time.

The active stress $s_n^{i,active}$ in each sector or compartment *n* at each timepoint *i* was then taken to be:

$$s_n^{i,active} = s_o \phi_n^i. \tag{4}$$

The elastic component of the stress depended on several assumptions. First, the cells were assumed to be in contact with ECM and other cells that were of the same mechanical properties, which is a reasonable approximation for cells at or near the percolation threshold [51]. Second, motivated by this assumption and one of symmetric boundary conditions, stress fibers were taken to have elastic stress proportional to their final stretch. Thus, before stretch was applied to the ETC, $s_n^{o,elastic} = \beta E(\lambda_n^o - 1)\phi_n^o$, where β is a term that relates ϕ to a volume fraction, and E is an elastic modulus appropriate for a stress fiber. After stretch, this elastic term was set to:

$$s_n^{elastic} = \beta E\left(\lambda_n^f - 1\right)\phi_n^1, \tag{5}$$

where ϕ_n^1 was the value immediately following stretch to model the case in which newly added stress fibers carry no elastic stress due to deformation of the cell, and λ_n^f is given by Equation (1).

In the simulations presented here, the following form of σ_n^i was taken:

$$\sigma_n^i = s_n^{elastic} + s_n^{i,active}. \tag{6}$$

In other simulations the effects of neighboring sectors or compartments were accounted for using $\sigma_n^i = \sum_{m=1}^{N} g_{mn}\left(s_m^{elastic} + s_m^{i,active}\right)$, where g_{mn} is a Green's function. This promoted reinforcement.

Finally, using a forward difference approximation for Equation (2), the time evolution of the fiber density in each compartment or sector was computed according to:

$$\phi_n^{i+1} = \phi_n^i + \frac{\Delta t}{\tau_G}\left(\phi_{max} - \phi_n^i\right) f\left(\sigma_n^i\right) \tag{7}$$

where Δt was taken as a fixed time increment. The time course of the normalized fibrosity metric was computed from these values.

Parameters used in simulations

The value used for βE was motivated by data in Figure S7 which showed the modulus of the ETC to be on the order of $E_{ETC}\approx 10$ kPa. Our earlier work suggested that $E_{ETC}\approx 10$ kPa is attained at a cell concentration associated with the percolation threshold for cells, and that at this cell concentration the effective moduli of cells and ECM are approximately identical [51]. βE in Equation (5) differs from E_{ETC} if the cell is taken not to resist shear deformation (that is, the stress fibers act as cables). Adjusting for this, with a circular model of the cell, yields an estimate of $\beta E \approx 25$ kPa. The threshold stress for stress fiber growth was set to $\sigma_G = 4s_o$, a value that required a significant deviation from the pre-stretch level of stress for stress-driven stress fiber growth.
