# Supplementary material for: Physically-Induced Cytoskeleton Remodeling of Cells in Three-Dimensional Culture
Source: PLoS One. 2012 Dec 27;7(12):e45512. doi: 10.1371/journal.pone.0045512 (PMC3531413; doi:10.1371/journal.pone.0045512)
Supplement: Discussion S1 — A simple example was studied to estimate the degree of passive changes to cell area and perimeter in response to stretch. This model was predicated on the observation that contractile fibroblasts and ECM have approximately the same elastic moduli in ETCs under the conditions tested, in which ETCs contain a population of cells just above the percolation threshold [51]. In this case, the strain in the local ECM should approximately equal that in the cells. (PDF) [file pone.0045512.s009.pdf]

Supplemental discussion: Effects of passive stretch on cell area and perimeter

Sheng-Lin Lee, et al., Physically-induced cytoskeleton remodeling of cells in three-dimensional culture.

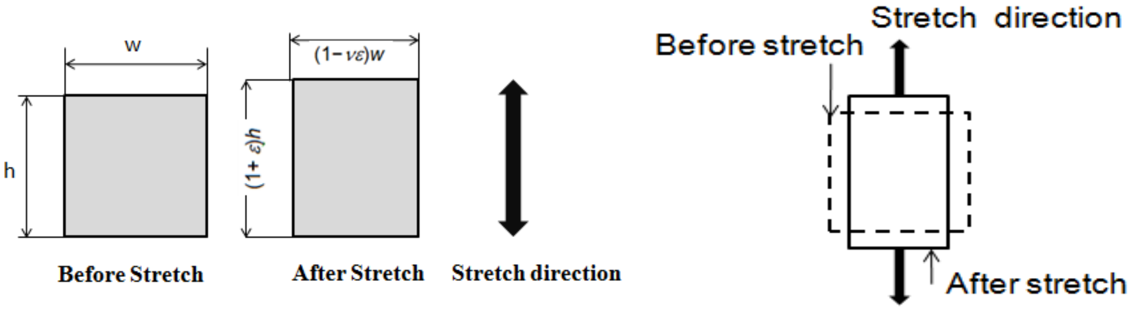

A simple example was studied to estimate the degree of passive changes to cell area and perimeter in response to stretch. This model was predicated on the observation that contractile fibroblasts and ECM have approximately the same elastic moduli in ETCs under the conditions tested, in which ETCs contain a population of cells just above the percolation threshold [51]. In this case, the strain in the local ECM should approximately equal that in the cells. For and idealized rectangular "cell" of width w and height h as in the above figure, the initial area can be estimated by assuming the area associated with filopodia-like extensions to be small: $A_o \approx wh$. Following stretch to a nominal strain ε, the cell's height grows to $(1+\varepsilon)h$ and its width shrinks to approximately $(1-\varepsilon\nu_{\mathrm{LT}})w$, where the Poisson ratio ν_{LT} relating transverse constriction to longitudinal extension of the area element was measured to be on the order of 0.9 to 1.1 by tracking the motion of fluorescent beads in the ECM. Then, the ratio of final to initial area is:

$$\frac{A}{A_o} = 1 + (1 - \nu_{LT})\epsilon - \nu_{LT}\epsilon^2.$$

For the strain range studied in this work, this yielded estimates of A/A_o between 0.98 and 1.00 for passive changes to cell area. Therefore, any significant deviations of area were indicative of active cellular remodeling, and not passive stretch.

Measurements of cell perimeter were more difficult to interpret because these must include the contributions of filopodia-like extensions. The perimeter measurement algorithm counts both sides of each filopodium, so the initial perimeter as reported through the algorithm is $p_o \approx 2\,(w + h + L_i)$, where L_i is the total pre-stretch length of all filopodia. Following stretch, the final perimeter is approximately:

$$p \approx 2\left((1 - \nu_{LT}\epsilon)w + (1 + \epsilon)h + L_f\right),$$

where L_f is the total post-stretch length of all filopodia. For a spindle-shaped cell, $h >> w$ and few filopodia are observed ($L_f \approx 0$), and

$$\frac{p}{p_o} \approx (1 + \epsilon).$$

For a stellate cell, $h \approx w$, and the contributions of filopodia are important. Then, following stretch,

$$\frac{p}{p_o} = \frac{2\left((1 - \nu_{LT}\epsilon)w + (1 + \epsilon)h + L_f\right)}{2\,(w + h + L_{\mathrm{i}})} \sim \frac{2 + (1 - \nu_{LT})\epsilon + L_f/h}{2 + L_{\mathrm{i}}/h} \sim \frac{2 + L_f/h}{2 + L_{\mathrm{i}}/h},$$

where the last step is true for v_{LT} close to 1. The perimeter measurements therefore represent predominantly changes in filopodial lengths in stellate cells. However, these are difficult to interpret in the context of cellular responses to stretch, because our observations suggested that retraction of cellular processes in the direction transverse to the direction of stretch were counterbalanced by extensions of cellular processes in the direction of stretch. Therefore, for both stellate and spindle-shaped cells, perimeter measurements proved less informative than area measurements for understanding temporal changes to cellular morphology.
